# Supplementary material for: Estimating individualized treatment rules in longitudinal studies with covariate-driven observation times
Source: Stat Methods Med Res. 2023 Mar 16;32(5):868–84. doi: 10.1177/09622802231158733 (PMC10248307; doi:10.1177/09622802231158733)
Supplement: sj-pdf-1-smm-10.1177_09622802231158733 - Supplemental material for Estimating individualized treatment rules in longitudinal studies with covariate-driven observation times [file sj-pdf-1-smm-10.1177_09622802231158733.pdf]

Supplementary Material for "Estimating Individualized Treatment Rules in Longitudinal Studies with Covariate-Driven Observation Times "

Janie Coulombe,[1] Erica E. M. Moodie, Susan M. Shortreed and Christel Renoux.

[1]Corresponding author: Department of Mathematics and Statistics, Université de Montréal, Pavillon André-Aisenstadt (AA-5190), 2920 chemin de la Tour, Montréal (Québec), H3T 1J4. Email: janie.coulombe@umontreal.ca.

Contents

Supplementary Materal A **2**

Supplementary Material B **4**

Supplementary Material C **6**

Supplementary Material D **10**

Supplementary Material E **11**

Supplementary Material F **17**

Supplementary Material G **18**

Supplementary Material H **21**

Supplementary Materal A

Supplementary Table 1: Summary of important notation and abbreviations used in this manuscript

Notation	Meaning	Abbreviation	Meaning
i	Patient index	BMI	Body mass index
n	Sample size	CI	Confidence interval
t	Time	CPRD	Clinical Practice Research Datalink
τ	Maximum follow-up time	DTR	Dynamic treatment regime
C_i	Censoring time	DWOLS	Dynamic weighted ordinary least squares
$\xi_i(t)$	Non-censoring indicator	DW	Doubly-weighted
$A_i(t)$	Binary treatment	EHR	Electronic health records
$Y_i(t)$	Continuous outcome	IIV	Inverse intensity of visit
$\mathbf{K}_i(t)$	Confounders	IPT	Inverse probability of treatment
$\mathbf{Q}_i(t)$	Tailoring variables	ITR	Individualized treatment rule
$\mathbf{X}^{\beta}(t)$	Risk factors for the outcome	MSE	Mean squared error
	excluding the treatment but	OLS	Ordinary least squares
	including a constant (intercept)	PS	Propensity score
$\mathbf{X}^{\psi}(t) = \mathbf{Q}_i(t)$	All risk factors for the outcome	SE	Standard error
	that also are tailoring variables	UK	United Kingdom
$\mathbf{X}(t) = \begin{bmatrix}\mathbf{X}^{\beta}(t) & \mathbf{X}^{\psi}(t)\end{bmatrix}$	Stacked matrix of predictors		
	that can be used as a design matrix		
$\mathbf{V}_i(t)$	Predictors of monitoring		
$\mathbf{Z}_i(t)$	Mediators of the treatment		
	effect that predict monitoring		
$N_i(t)$	Monitoring counting process		
$dN_i(t)$	Monitoring indicator		
$Y_i^a(t)$	Potential outcome under treatment $A = a$		
$\Lambda_0(t)$	Cumulative baseline rate		
$\boldsymbol{\beta}$	Parameters of the		
	treatment-free model		
$\boldsymbol{\psi}$	Parameters of the blip model		
$\boldsymbol{\gamma}$	Parameters of the monitoring		
	model		
$\boldsymbol{\kappa}$	Parameters of the propensity		
	score model		
$w(\cdot)$	The IPT weight function		
$\rho(\cdot)$	The IIV weight function		
$\hat{\boldsymbol{\psi}}_{OLS}$	Blip estimator that uses none of the		
	IPT or IIV weights		
$\hat{\boldsymbol{\psi}}_{IPT}$	Blip estimator that uses		
	the IPT weight but not the IIV weight		
$\hat{\boldsymbol{\psi}}_{DW1}$	Proposed doubly-weighted blip		
	estimator with correctly specified treatment (PS)		
	and monitoring models		
$\hat{\boldsymbol{\psi}}_{DW2}$	Proposed doubly-weighted blip		
	estimator with partially misspecified monitoring		
	and misspecified outcome models		
$\hat{\boldsymbol{\psi}}_{DW3}$	Proposed doubly-weighted blip		
	estimator with partially misspecified monitoring		
	and misspecified treatment (PS) models		
$\hat{\boldsymbol{\psi}}_{DW4}$	Proposed doubly-weighted blip		
	estimator with misspecified monitoring		
	model		
$U(t)$	Outcome (utility function) in the		
	illustration to CPRD		

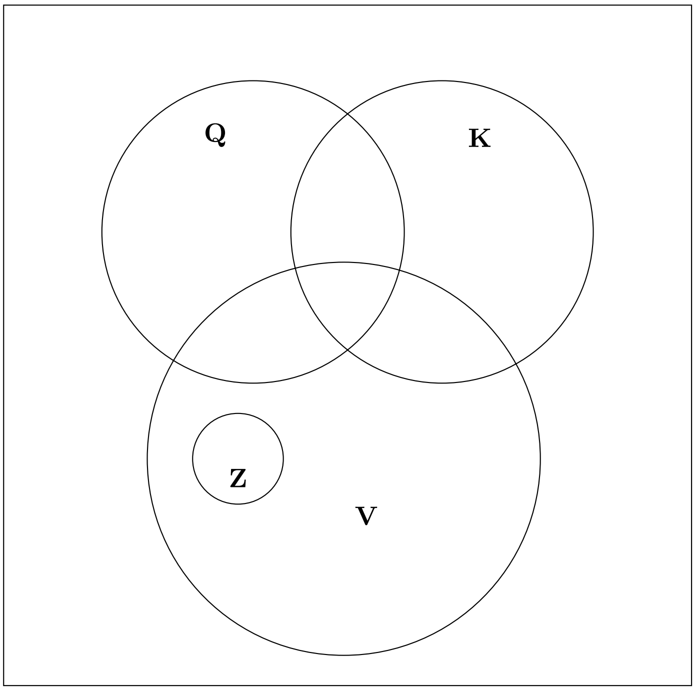

Supplementary Figure 1: Venn diagram to visualize the potential overlaps between the different sets of covariates.

Supplementary Table 2: Models used in the manuscript with their number

Number	Name	Model
(V1)	Observation model (proportional rate model)	$\xi_i(t)\exp\{\boldsymbol{\gamma}'\mathbf{V}_i(t)\}\,d\Lambda_0(t) \underset{\mathbf{V}}{\propto} \xi_i(t)\exp\{\boldsymbol{\gamma}'\mathbf{V}_i(t)\}$
None	Treatment model (logistic regression model)	$P(A_i(t) \mid \mathbf{K}_i(t);\boldsymbol{\kappa}) = \frac{\mathbb{I}(A_i(t)=1)\exp^{\boldsymbol{\kappa}'\mathbf{K}_i(t)}}{1+\exp^{\boldsymbol{\kappa}'\mathbf{K}_i(t)}}$
None	Propensity score (treatment model evaluated at 1)	$P(A_i(t)=1 \mid \mathbf{K}_i(t);\boldsymbol{\kappa}) = \frac{\exp^{\boldsymbol{\kappa}'\mathbf{K}_i(t)}}{1+\exp^{\boldsymbol{\kappa}'\mathbf{K}_i(t)}}$
⊂(O2)	Treatment-free model (part of the outcome model)	$f\left\{\mathbf{X}_i^{\beta}(t);\boldsymbol{\beta}\right\} = \boldsymbol{\beta}'\mathbf{X}_i^{\beta}(t)$
⊂(O2)	Blip model (part of the outcome model)	$\boldsymbol{\psi}'\mathbf{X}_i^{\psi}(t)$
(O2)	Mean outcome model	$f\left\{\mathbf{X}_i^{\beta}(t);\boldsymbol{\beta}\right\} + A_i(t)\boldsymbol{\psi}'\mathbf{X}_i^{\psi}(t)$

Supplementary Material B

Supplementary Figure 2 presents in (a) the data generating mechanism in simulation studies at time t. Note, the individual index is removed for ease of notation and interactions are not depicted in any diagrams in Supplementary Figure 2. Panels (b) to (g) show the associations remaining after using the weights of the corresponding estimators: (b) $\hat{\psi}_{DW1}$ (all models correctly specified); (c) $\hat{\psi}_{DW2}$ (partially misspecified observation model w.r.t. K_2 and K_3, and misspecified outcome model w.r.t. K_2); (d) $\hat{\psi}_{DW3}$ (partially misspecified observation model w.r.t. K_2 and K_3, and misspecified treatment model w.r.t. K_1 and K_3); (e) $\hat{\psi}_{DW4}$ (misspecified observation model w.r.t. $Z(t)$ and K_3); (f) $\hat{\psi}_{IPT}$ (no adjustment for the observation model); (g) $\hat{\psi}_{OLS}$ (no adjustment for the observation model and no adjustment for the treatment model via an IPT weight). A box represents conditioning on the corresponding variable in the mean outcome model for all variables except the observation indicator $dN(t)$, which is implicitly conditioned upon by virtue of estimation relying only on observed data. A dashed line represents a relationship that is possibly remaining due to a misspecified model. For figures (a), (e), (f) and (g) we find a path (an association) remaining that goes from $A(t)$ to $dN(t)$ to $Z(t)$ to $Y(t)$ that is not due to the causal effect of $A(t)$. The observation model adjusting only for $A(t)$ and K_2 is misspecified w r.t. to $Z(t)$ and K_3, but, as discussed in the main manuscript, it is also possibly misspecified with respect to $A(t)$ since that variable is associated with $Z(t)$. The coefficient for $A(t)$ in the observation model may, therefore, be biased in the subadjusted model containing only $A(t)$ and K_2.

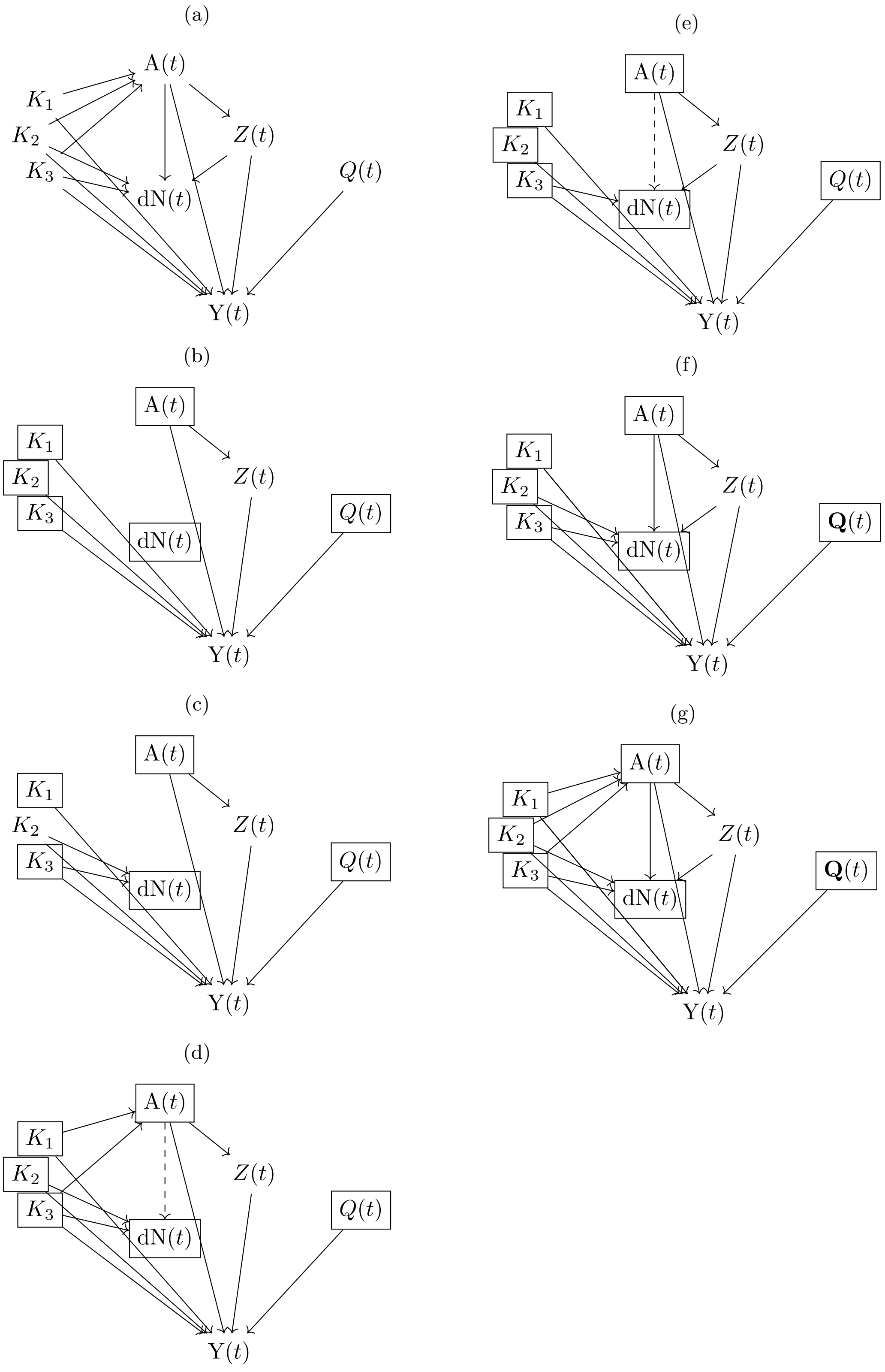

Supplementary Figure 2: (a) The data generating mechanism in simulations. Panels (b) to (g) show the associations remaining after using the weights of the corresponding estimators (and boxes are used to represent the variables upon which we condition in each mean outcome model or, in the case of the observation indicator $dN(t)$, that we implicitly condition upon by using only observed data): (b) $\hat{\psi}_{DW1}$; (c) $\hat{\psi}_{DW2}$; (d) $\hat{\psi}_{DW3}$; (e) $\hat{\psi}_{DW4}$; (f) $\hat{\psi}_{IPT}$; and (g) $\hat{\psi}_{OLS}$. A dashed line represents a relationship that is possibly remaining due to a misspecified observation model (either an observation model lacking predictors, or for which some parameters are estimated with bias because of other dependent predictors missing in the model).

Supplementary Material C

Simulation study results: error rate (i.e., empirical MSE) of the estimated optimal treatment decisions, absolute empirical bias of the blip values, absolute bias of each coefficient in the blip function, and average estimated value function, as obtained from the six alternative estimators

Supplementary Table 3: Simulation study results ($M = 1000$ simulations) for the comparison of error rate of the estimated optimal **treatment decision** obtained with six alternative models: DW1 the proposed doubly-weighted estimator which accounts for both processes correctly, DW2 for which the observation process was partially misspecified and the outcome model was misspecified, DW3 for which the treatment process was misspecified and the observation process was partially misspecified, DW4 for which the observation process was misspecified, OLS which does not adjust for confounding or observation process, and IPW which accounts only for confounding. Empirical MSEs are computed as the squared empirical bias of the estimated optimal treatment decision (based on the estimated blip function) plus its empirical variance. The observation process varies but the confounding mechanism and the parameters of the true blip function remain the same in all 4 scenarios of varying γ below.

Sample size	γ^{υ} parameters	No. obs. times mean (IQR)	Error rate $\hat{\psi}_{DW1}$	$\hat{\psi}_{DW2}$	$\hat{\psi}_{DW3}$	$\hat{\psi}_{DW4}$	$\hat{\psi}_{OLS}$	$\hat{\psi}_{IPT}$
250	1	3 (1-3)	0.02	0.01	0.01	0.04	0.03	0.04
	2	3 (2-5)	0.05	0.06	0.05	0.16	0.15	0.16
	3	6 (3-9)	0.06	0.03	0.03	0.26	0.25	0.26
	4	10 (8-12)	0.01	0.01	0.00	0.01	0.00	0.01
500	1	3 (1-3)	0.01	0.01	0.01	0.03	0.03	0.03
	2	3 (1-5)	0.02	0.03	0.02	0.14	0.13	0.14
	3	6 (3-9)	0.04	0.02	0.02	0.25	0.25	0.25
	4	10 (8-12)	0.00	0.00	0.00	0.00	0.00	0.00

υ.1. (-2, -0.3, 0.2, -1.2); 2. (0.3, -0.6, -0.4, -0.3); 3. (0.4, -0.8, 1, 0.6); 4. (0, 0, 0, 0), i.e., uninformative observation.
Abbreviations: MSE, mean squared error; IQR, interquartile range.

Supplementary Table 4: Simulation study results ($M = 1000$ simulations) for the comparison of **absolute bias** of the **blip values** obtained with six alternative models: DW1 the proposed doubly-weighted estimator which accounts for both processes correctly, DW2 for which the observation process was partially misspecified and the outcome model was misspecified, DW3 for which the treatment process was misspecified and the observation process was partially misspecified, DW4 for which the observation process was misspecified, OLS which does not adjust for confounding or observation process, and IPW which accounts only for confounding. The observation process varies but the confounding mechanism and the parameters of the true blip function remain the same in all 4 scenarios of varying $\boldsymbol{\gamma}$ below.

Sample size	$\boldsymbol{\gamma}^{\upsilon}$ parameters	Mean no. obs. times (IQR)	Absolute bias $\hat{\boldsymbol{\psi}}_{DW1}$	$\hat{\boldsymbol{\psi}}_{DW2}$	$\hat{\boldsymbol{\psi}}_{DW3}$	$\hat{\boldsymbol{\psi}}_{DW4}$	$\hat{\boldsymbol{\psi}}_{OLS}$	$\hat{\boldsymbol{\psi}}_{IPT}$
250	1	3 (1-3)	0.58	0.50	0.42	0.76	0.74	0.76
	2	3 (1-5)	1.00	1.07	0.97	1.57	1.53	1.57
	3	6 (3-9)	1.14	0.87	0.83	2.04	2.06	2.06
	4	10 (8-12)	0.24	0.24	0.21	0.24	0.19	0.24
500	1	3 (1-3)	0.44	0.37	0.30	0.70	0.73	0.69
	2	3 (1-5)	0.73	0.80	0.70	1.54	1.53	1.54
	3	6 (3-9)	0.89	0.65	0.63	2.04	2.05	2.05
	4	10 (8-12)	0.17	0.17	0.15	0.17	0.13	0.17
1000	1	3 (1-3)	0.33	0.27	0.21	0.68	0.72	0.67
	2	3 (1-5)	0.55	0.60	0.53	1.50	1.49	1.50
	3	6 (3-9)	0.69	0.48	0.46	2.03	2.04	2.04
	4	10 (8-12)	0.12	0.12	0.10	0.12	0.09	0.12
2500	1	3 (1-3)	0.23	0.18	0.14	0.66	0.71	0.66
	2	3 (2-5)	0.36	0.40	0.35	1.51	1.51	1.50
	3	6 (3-9)	0.52	0.34	0.32	2.04	2.05	2.05
	4	10 (8-12)	0.08	0.08	0.07	0.08	0.06	0.08

υ.1. (-2, -0.3, 0.2, -1.2); 2. (0.3, -0.6, -0.4, -0.3); 3. (0.4, -0.8, 1, 0.6); 4. (0, 0, 0, 0), i.e., uninformative observation.
Abbreviations: MSE, mean squared error; IQR, interquartile range.

Supplementary Table 5: Simulation study results ($M = 1000$ simulations) for the comparison of **absolute bias** of the **coefficients** (standard error of the coefficient in parentheses) in the blip function obtained with six alternative models: DW1 the proposed doubly-weighted estimator which accounts for both processes correctly, DW2 for which the observation process was partially misspecified and the outcome model was misspecified, DW3 for which the treatment process was misspecified and the observation process was partially misspecified, DW4 for which the observation process was misspecified, OLS which does not adjust for confounding or observation process, and IPW which accounts only for confounding. The observation process varies but the confounding mechanism and the parameters of the true blip function remain the same in all 4 scenarios of varying $\boldsymbol{\gamma}$ below.

$\boldsymbol{\gamma}^{\upsilon}$	Estimator		$n = 250$			$n = 500$	
parameters	for the ITR	Intercept	K_1	Q	Intercept	K_1	Q
1	$\hat{\boldsymbol{\psi}}_{DW1}$	0.09 (0.75)	0.01 (0.86)	0.03 (0.46)	0.09 (0.56)	0.01 (0.65)	0.02 (0.34)
	$\hat{\boldsymbol{\psi}}_{DW2}$	0.02 (0.60)	0.01 (0.75)	0.04 (0.40)	0.05 (0.44)	0.00 (0.55)	0.02 (0.29)
	$\hat{\boldsymbol{\psi}}_{DW3}$	0.02 (0.50)	0.02 (0.62)	0.02 (0.33)	0.02 (0.35)	0.00 (0.44)	0.01 (0.23)
	$\hat{\boldsymbol{\psi}}_{DW4}$	0.69 (0.53)	0.00 (0.67)	0.00 (0.36)	0.68 (0.40)	0.00 (0.50)	0.01 (0.26)
	$\hat{\boldsymbol{\psi}}_{OLS}$	0.74 (0.42)	0.00 (0.52)	0.01 (0.25)	0.73 (0.30)	0.00 (0.37)	0.00 (0.18)
	$\hat{\boldsymbol{\psi}}_{IPT}$	0.69 (0.53)	0.00 (0.67)	0.00 (0.36)	0.67 (0.40)	0.00 (0.49)	0.01 (0.26)
2	$\hat{\boldsymbol{\psi}}_{DW1}$	0.28 (1.09)	0.01 (1.39)	0.12 (0.67)	0.13 (0.84)	0.03 (1.03)	0.09 (0.54)
	$\hat{\boldsymbol{\psi}}_{DW2}$	0.37 (1.10)	0.02 (1.40)	0.12 (0.68)	0.21 (0.87)	0.05 (1.07)	0.10 (0.57)
	$\hat{\boldsymbol{\psi}}_{DW3}$	0.32 (1.06)	0.02 (1.33)	0.06 (0.61)	0.14 (0.80)	0.03 (0.97)	0.07 (0.48)
	$\hat{\boldsymbol{\psi}}_{DW4}$	1.51 (0.83)	0.03 (1.07)	0.01 (0.59)	1.52 (0.62)	0.01 (0.78)	0.00 (0.43)
	$\hat{\boldsymbol{\psi}}_{OLS}$	1.50 (0.69)	0.03 (0.86)	0.01 (0.46)	1.52 (0.48)	0.01 (0.58)	0.00 (0.32)
	$\hat{\boldsymbol{\psi}}_{IPT}$	1.51 (0.84)	0.03 (1.08)	0.00 (0.60)	1.52 (0.61)	0.01 (0.79)	0.01 (0.43)
3	$\hat{\boldsymbol{\psi}}_{DW1}$	0.50 (1.21)	0.13 (1.40)	0.13 (0.72)	0.23 (0.96)	0.05 (1.14)	0.10 (0.60)
	$\hat{\boldsymbol{\psi}}_{DW2}$	0.22 (1.03)	0.09 (1.17)	0.09 (0.62)	0.06 (0.74)	0.01 (0.91)	0.07 (0.48)
	$\hat{\boldsymbol{\psi}}_{DW3}$	0.24 (0.98)	0.08 (1.08)	0.07 (0.56)	0.05 (0.73)	0.01 (0.86)	0.07 (0.44)
	$\hat{\boldsymbol{\psi}}_{DW4}$	2.05 (0.57)	0.00 (0.72)	0.01 (0.38)	2.03 (0.40)	0.02 (0.50)	0.00 (0.27)
	$\hat{\boldsymbol{\psi}}_{OLS}$	2.05 (0.43)	0.01 (0.49)	0.00 (0.27)	2.03 (0.30)	0.02 (0.35)	0.00 (0.19)
	$\hat{\boldsymbol{\psi}}_{IPT}$	2.06 (0.50)	0.00 (0.62)	0.01 (0.34)	2.04 (0.36)	0.01 (0.44)	0.00 (0.24)
4	$\hat{\boldsymbol{\psi}}_{DW1}$	0.00 (0.29)	0.00 (0.38)	0.00 (0.20)	0.01 (0.21)	0.01 (0.26)	0.00 (0.14)
	$\hat{\boldsymbol{\psi}}_{DW2}$	0.00 (0.29)	0.00 (0.38)	0.00 (0.20)	0.01 (0.21)	0.01 (0.26)	0.00 (0.14)
	$\hat{\boldsymbol{\psi}}_{DW3}$	0.00 (0.24)	0.01 (0.30)	0.00 (0.17)	0.01 (0.17)	0.00 (0.21)	0.00 (0.12)
	$\hat{\boldsymbol{\psi}}_{DW4}$	0.00 (0.30)	0.00 (0.38)	0.00 (0.20)	0.01 (0.21)	0.01 (0.26)	0.00 (0.14)
	$\hat{\boldsymbol{\psi}}_{OLS}$	0.00 (0.23)	0.01 (0.29)	0.00 (0.14)	0.01 (0.16)	0.00 (0.19)	0.00 (0.10)
	$\hat{\boldsymbol{\psi}}_{IPT}$	0.00 (0.30)	0.00 (0.38)	0.00 (0.20)	0.01 (0.21)	0.01 (0.26)	0.00 (0.14)

υ.1. (-2, -0.3, 0.2, -1.2); 2. (0.3, -0.6, -0.4, -0.3); 3. (0.4, -0.8, 1, 0.6); 4. (0, 0, 0, 0), i.e., uninformative observation.

Supplementary Table 6: Simulation study results ($M = 1000$ simulations, $n = 25,000$) of the **estimated value function** using the true data generating mechanism for all other variables than the treatment, and a treatment either based on the true data generating mechanism or on six alternative optimal treatment decisions: DW1 the proposed doubly-weighted estimator which accounts for both processes correctly, DW2 for which the observation process was partially misspecified and the outcome model was misspecified, DW3 for which the treatment process was misspecified and the observation process was partially misspecified, DW4 for which the observation process was misspecified, OLS which does not adjust for confounding or observation process, and IPW which accounts only for confounding. The observation process varies but the confounding mechanism and the parameters of the true blip function remain the same in all 4 scenarios of varying $\boldsymbol{\gamma}$ below.

	Estimated value function in the large dataset						
$\boldsymbol{\gamma}^{\upsilon}$	Actual treatment	$\hat{\boldsymbol{\psi}}^{\dagger}_{DW1}$	$\hat{\boldsymbol{\psi}}^{\dagger}_{DW2}$	$\hat{\boldsymbol{\psi}}^{\dagger}_{DW3}$	$\hat{\boldsymbol{\psi}}^{\dagger}_{DW4}$	$\hat{\boldsymbol{\psi}}^{\dagger}_{OLS}$	$\hat{\boldsymbol{\psi}}^{\dagger}_{IPT}$
1	-1.05	0.54	0.55	0.55	0.52	0.53	0.52
2	-2.86	-0.31	-0.32	-0.30	-0.49	-0.45	-0.50
3	-3.82	-1.34	-1.29	-1.29	-1.63	-1.61	-1.62
4	-0.86	1.10	1.10	1.10	1.10	1.10	1.10

υ. 1. (-2, -0.3, 0.2, -1.2); 2. (0.3, -0.6, -0.4, -0.3); 3. (0.4, -0.8, 1, 0.6); 4. (0, 0, 0, 0), i.e., uninformative observation.
†. Under the optimal treatment (as per the corresponding estimated optimal ITR).

Supplementary Material D

Results of the sensitivity analysis with a sample size of 50,000 patients (comparison with the results under a sample size of 250)

Supplementary Table 7: Sensitivity analysis - Simulation study results for the comparison of **absolute bias** of the **coefficients** (standard error of the coefficient in parentheses) in the blip function obtained with six alternative models: DW1 the proposed doubly-weighted estimator which accounts for both processes correctly, DW2 for which the observation process was partially misspecified and the outcome model was misspecified, DW3 for which the treatment process was misspecified and the observation process was partially misspecified, DW4 for which the observation process was misspecified, OLS which does not adjust for confounding or observation process, and IPW which accounts only for confounding. The observation process varies but the confounding mechanism and the parameters of the true blip function remain the same in all 4 scenarios of varying $\boldsymbol{\gamma}$ below.

$\boldsymbol{\gamma}^{v}$	Estimator	$n = 250$, $M = 1000$ simulations			$\mathbf{n = 50,000}$, $M = 200$ simulations		
parameters	for the ITR	Intercept	K_1	Q	Intercept	K_1	Q
1	$\hat{\boldsymbol{\psi}}_{DW1}$	0.09 (0.75)	0.01 (0.86)	0.03 (0.46)	0.05 (0.07)	0.01 (0.08)	0.00 (0.05)
	$\hat{\boldsymbol{\psi}}_{DW2}$	0.02 (0.60)	0.01 (0.75)	0.04 (0.40)	0.07 (0.05)	0.00 (0.06)	0.00 (0.03)
	$\hat{\boldsymbol{\psi}}_{DW3}$	0.02 (0.50)	0.02 (0.62)	0.02 (0.33)	0.03 (0.03)	0.00 (0.04)	0.00 (0.03)
	$\hat{\boldsymbol{\psi}}_{DW4}$	0.69 (0.53)	0.00 (0.67)	0.00 (0.36)	0.67 (0.04)	0.00 (0.05)	0.00 (0.02)
	$\hat{\boldsymbol{\psi}}_{OLS}$	0.74 (0.42)	0.00 (0.52)	0.01 (0.25)	0.72 (0.03)	0.00 (0.03)	0.00 (0.02)
	$\hat{\boldsymbol{\psi}}_{IPT}$	0.69 (0.53)	0.00 (0.67)	0.00 (0.36)	0.67 (0.04)	0.00 (0.05)	0.00 (0.02)
3	$\hat{\boldsymbol{\psi}}_{DW1}$	0.50 (1.21)	0.13 (1.40)	0.13 (0.72)	0.18 (0.22)	0.02 (0.28)	0.02 (0.13)
	$\hat{\boldsymbol{\psi}}_{DW2}$	0.22 (1.03)	0.09 (1.17)	0.09 (0.62)	0.08 (0.10)	0.01 (0.13)	0.01 (0.07)
	$\hat{\boldsymbol{\psi}}_{DW3}$	0.24 (0.98)	0.08 (1.08)	0.07 (0.56)	0.08 (0.10)	0.01 (0.12)	0.01 (0.07)
	$\hat{\boldsymbol{\psi}}_{DW4}$	2.05 (0.57)	0.00 (0.72)	0.01 (0.38)	2.03 (0.04)	0.00 (0.05)	0.00 (0.03)
	$\hat{\boldsymbol{\psi}}_{OLS}$	2.05 (0.43)	0.01 (0.49)	0.00 (0.27)	2.04 (0.03)	0.00 (0.04)	0.00 (0.02)
	$\hat{\boldsymbol{\psi}}_{IPT}$	2.06 (0.50)	0.00 (0.62)	0.01 (0.34)	2.05 (0.04)	0.00 (0.05)	0.00 (0.03)

v.1. (-2, -0.3, 0.2, -1.2); 3. (0.4, -0.8, 1, 0.6).

Supplementary Material E

Sample of R code to reproduce the analysis (i.e., to apply the estimator) and running times

Note. This code is also available at `https://github.com/janiecoulombestat` under the repository `IIV-ATS project`. At the end of the R code below, you will find the time required to run one simulation, separately by different steps (i.e., create one dataset, fit the weight models once, and run the analyses once) for different sample sizes. These times can roughly be multiplied by 1000 to know the time required to run 1000 simulations (as was done in the main manuscript).

```
###############################
## A first version of this R code was written by Janie Coulombe
## and it was made more efficient
##  by an anonymous reviewer
##
## For manuscript entitled
## 'Estimating individualized treatment rules in longitudinal studies
   ## with covariate-driven observation times'
## Co-authors Erica EM Moodie, Susan M Shortreed, Christel Renoux
## October 2022
###############################



rm(list=ls(all=TRUE))

## Packages to download
library(survival)
library(splines)

ss <- function()source("simul_new.r")
sss <- function(){ss(); simul_new()}

# FUNCTION SIMUL, can be used to simulate the datasets and to estimate the blip
# function (i.e., its coefficients)
# using the 6 different estimators
# VH:
# LOOP-FREE data generation

#-- simul.new ------------------------------------------------------------

simul.new<- function(sampsize=250, nbsimul=2 ,print=F, gammaset=3){

# Change according to sample size (sampsize) and nb of simulations (nsbimul)
# desired print=TRUE shows the time needed to perform only the analysis part
# (fit the regression models, etc.) for DW1 estimator
# VH:
# gammaset=<i> : parameters <i> of Table 1 is applied (1, 2, 3 or 4).
# global parameters below now start with ".."

## Maximum follow-up time TAU
    TAU<-1

## Parameters outcome model
    # K1           K2           K3
    ..beta4<-0.4; ..beta5<-0.05; ..beta6<- -0.6;
    # Q*treatm      K1*treatm     treatment
    ..bint<- 0.5; ..bint2<- -1; ..betaA<- -2
```

```
## For error term in outcome model
    ..sigmaepsilond<- 0.1; ..meanphid=0; ..sigmaphid=0.2;

## Parameters related to mediator Z
    ..mu1d=4; ..sigma2_1d=2; ..mu2d=2; ..sigma2_2d=1; ..betaZ<- 2.5

## Treatment model parameters
    ..beta0=0.5; ..beta1=0.55; ..beta2=-0.2; ..beta3=-1;

# For defining the 3 confounders K1, K2, K3
    ..K1mean=1; ..K1sd=1; ..p_K2=0.55; ..K3mean=0; ..K3sd=1;

## Outcome observation model (gamma parameters)
    #               A                Z              K2            K3
    if (gammaset==1){
        ..gamma1<- -2.0; ..gamma2<- -0.3; ..gamma3<-  0.2; ..gamma4<- -1.2}
    if (gammaset==2){
        ..gamma1<-  0.3; ..gamma2<- -0.6; ..gamma3<- -0.4; ..gamma4<- -0.3}
    if (gammaset==3){
        ..gamma1<-  0.4; ..gamma2<- -0.8; ..gamma3<-  1.0; ..gamma4<-  0.6}
    if (gammaset==4){
        ..gamma1<-    0; ..gamma2<-    0; ..gamma3<-    0; ..gamma4<-    0}

    # data.frames to store the results, return at end of function
    coefMat <- data.frame(matrix(nr=nbsimul, nc=3*6))
    names(coefMat) <- c("DW1_A","DW1_K1","DW1_Q"
                        ,"DW2_A","DW2_K1","DW2_Q"
                        ,"DW3_A","DW3_K1","DW3_Q"
                        ,"DW4_A","DW4_K1","DW4_Q"
                        ,"OLS_A","OLS_K1","OLS_Q"
                        ,"IPT_A","IPT_K1","IPT_Q"
                        )
    MSEmat <- data.frame(matrix(nr=nbsimul, nc=7))
    names(MSEmat) <- c("DW1","DW2","DW3","DW4","OLS","IPT","meanOBS")

    visitMat <- data.frame(matrix(nr=nbsimul, nc=3))
    names(visitMat) <- c("mVisit","mAtot","mAVisit")

    # -------------------------------------------------------------------
    # start loop over simulations
    # -------------------------------------------------------------------
    for(S in 1: nbsimul){
        start_time1 <- Sys.time()
        if (S<5||S%%(ceiling(nbsimul/20))==0) cat("vh:",S, fill=T)

        # ===================================================================
        # start data generation (mat) in a non-loop manner

        N.pers <- sampsize
        N.times <- TAU*100
        N.tot <- N.pers*N.times
        mat<- data.frame(matrix(NA, nrow=N.tot, ncol= 11))
        names(mat)<-
        c('ID','time','A','Y','K1','K2','K3','Qvar','Fother','visit','Z')
        #  1    2      3   4   5    6    7    8      9        10      11

        mat[,'ID']     <- rep(1:N.pers, each=N.times)
        mat[,'time']   <- rep(1:N.times/100,   N.pers)
```

```
mat[,'K1']    <-
    rep(rnorm(mean=..K1mean,    sd=..K1sd,    n=N.pers),each=N.times)
mat[,'K2']    <-
    rep(rbinom(p=..p_K2,         size=1,          n=N.pers),each=N.times)
mat[,'K3'] <-
    rep(rnorm(mean=..K3mean, sd=..K3sd, n=N.pers),each=N.times)

p_treated <-
   exp(..beta0+ ..beta1*mat$K1+ ..beta2*mat$K2+ ..beta3*mat$K3)/
(1+exp(..beta0+ ..beta1*mat$K1+ ..beta2*mat$K2+ ..beta3*mat$K3))
mat[,'A']      <- rbinom(p=p_treated, size=1, n=N.tot)

Z1a <- rnorm(mean=..mu1d, sd=sqrt(..sigma2_1d), n=N.tot) ## N(4,2)
Z1b <- rnorm(mean=..mu2d, sd=sqrt(..sigma2_2d), n=N.tot) ## N(2,1)
mat[,'Z']      <- ifelse(mat$A==1,Z1b,Z1a)

mat[,'Qvar']   <- rnorm(mean=0.5, sd=0.5, n=N.tot)

alpha0  <-rep(sqrt(1:N.times/100),                  N.pers)
phi     <-rnorm(mean=..meanphid, sd=..sigmaphid,     n=N.tot)
epsilon <-rnorm(mean=phi        , sd=..sigmaepsilond, n=N.tot)
center  <-ifelse(mat$A==1, ..mu2d, ..mu1d)  # 2, 4
mat[,'Y'] <- alpha0 +
       ..betaA*mat$A +
       ..beta4*mat$K1 + ..beta5*mat$K2 + ..beta6*mat$K3 +
       ..betaZ*(mat$Z-center) +
       ..bint *mat$A*mat$Qvar +
       ..bint2*mat$A*mat$K1  + epsilon

mat[,'Fother'] <- rbinom(p= 0.5, size=1, n=N.tot)

ratei<-  exp(..gamma1*mat[,'A']   + ..gamma2*mat[,'Z']   +
             ..gamma3*mat[,'K2'] + ..gamma4*mat[,'K3'] )*0.1
ratei[ratei>1]<-1 # remove rates higher than 1 as a probability of jump
# over tiny time increment

mat[,'visit']<-  rbinom(prob= ratei, size=1, n=N.tot)

start_time2 <- Sys.time()
if(print=='TRUE'&&S==1){ # print if first simulation
    cat("loop:");print(start_time2-start_time1)}# VH

# data generation finished
# ==========================================================================
############################################
## Compute weights                        ##
############################################
start_time2 <- Sys.time()

## Keep only the visits
Data <- mat
Data[Data$visit==0,]$Y<-NA ## Put outcome as missing if no visit

## Visit intensity model (need counting process format)
Data$t1<-Data$time-0.01
Data$t2<-Data$time
```

```
gamma  <-coxph(Surv(t1,t2,visit)~A+Z+K2+K3, data=Data)$coef
## 2 other models, for the wrongly specified visit models:
gamma2b<-coxph(Surv(t1,t2,visit)~A+Z          , data=Data)$coef
gamma3b<-coxph(Surv(t1,t2,visit)~A  +K2       , data=Data)$coef

## right model:
Data$rho_i<-     exp(gamma  [1]*Data$A +   gamma[2]*Data$Z +
                         gamma  [3]*Data$K2 + gamma[4]*Data$K3)
## wrong model but no expected bias:
Data$rho_i2<-    exp(gamma2b[1]*Data$A + gamma2b[2]*Data$Z)

## wrong model and expected bias:
Data$rho_i3<-       exp(gamma3b[1]*Data$A + gamma3b[2]*Data$K2)

# Compute the propensity scores and two types of IPTW (one correct
# iptw, one wrong iptw2):
ps <-predict(glm(A ~ K1 + K2 + K3,
    data=Data, family='binomial'),type='response')

Data$iptw <- 1/ifelse(Data$A==1, ps , (1-ps ))

ps2<-predict(glm(A ~ I(K1^2) + K2 + I(sin(K3^2)),
    data=Data, family='binomial'),type='response')
Data$iptw2<- 1/ifelse(Data$A==1, ps2, (1-ps2))

start_time3 <- Sys.time() # VH
if(print=='TRUE'&&S==1){
    cat("ps:  ");print(start_time3-start_time2)}# VH

############################################
## Compute the different blip functions   ##
############################################
## DOUBLY WEIGHTED DW1 ##
#           1      2     3     4   5     6      7     8        9     10      11
# intercept, bs1, bs2, bs3, A, K1, Qvar, K2, K3, A:K1, A:Qvar
coefs <- lm(Y~bs(time,degree=3)+ A + K1*A + K1 + Qvar + Qvar*A + K2 +
    K3, weight=iptw *1/rho_i  , data=Data)$coef
coef_DW1_A   <-coefs[5]
coef_DW1_K1 <-coefs[10]
coef_DW1_Q   <-coefs[11]

## DOUBLY WEIGHTED DW2 ## K2 missing, wrong IIV weight
coefs <-lm(Y~bs(time,degree=3) + A + K1*A + K1 + Qvar + Qvar*A         +
   K3, weight=iptw *1/rho_i2, data=Data)$coef
coef_DW2_A   <-coefs[5]
coef_DW2_K1 <-coefs[9]
coef_DW2_Q   <-coefs[10]

## DOUBLY WEIGHTED DW3 ##  wrong IIV and IPT weight
coefs <-lm(Y~bs(time,degree=3) + A + K1*A + K1 + Qvar + Qvar*A + K2 +
    K3, weight=iptw2*1/rho_i2, data=Data)$coef
coef_DW3_A   <-coefs[5]
coef_DW3_K1 <-coefs[10]
coef_DW3_Q   <-coefs[11]

## DOUBLY WEIGHTED DW4 ## wrong IIV weight
coefs <-lm(Y~bs(time,degree=3) + A + K1*A + K1 + Qvar + Qvar*A + K2 +
    K3, weight=iptw *1/rho_i3, data=Data)$coef
```

```
    coef_DW4_A    <-coefs[5]
    coef_DW4_K1 <-coefs[10]
    coef_DW4_Q    <-coefs[11]

  ## OLS ESTIMATOR ## observation prosess ignored
    coefs <-lm(Y~bs(time,degree=3) + A + K1*A + K1 + Qvar + Qvar*A + K2 +
       K3                        , data=Data)$coef
    coef_OLS_A    <-coefs[5]
    coef_OLS_K1 <-coefs[10]
    coef_OLS_Q    <-coefs[11]

  ## IPT ESTIMATOR ## observation prosess ignored,
    coefs <-lm(Y~bs(time,degree=3) + A + K1*A + K1 + Qvar + Qvar*A + K2 +
        K3, weight=iptw             , data=Data)$coef
    coef_IPT_A    <-coefs[5]
    coef_IPT_K1 <-coefs[10]
    coef_IPT_Q    <-coefs[11]

  ## OLS ESTIMATOR ## K2 K3 totally left out
    coefs <-lm(Y~bs(time,degree=3) + A + K1*A + K1 + Qvar + Qvar*A
                                    , data=Data)$coef
    coef_OLS0_A    <-coefs[5]
    coef_OLS0_K1 <-coefs[8]
    coef_OLS0_Q    <-coefs[9]

    end_time4 <- Sys.time()    # VH
    if(print=='TRUE'&&S==1){
        cat("glm: ");print(end_time4-start_time3) }

    coefMat[S,] <- c(coef_DW1_A, coef_DW1_K1, coef_DW1_Q
                    ,coef_DW2_A, coef_DW2_K1, coef_DW2_Q
                    ,coef_DW3_A, coef_DW3_K1, coef_DW3_Q
                    ,coef_DW4_A, coef_DW4_K1, coef_DW4_Q
                    ,coef_OLS_A, coef_OLS_K1, coef_OLS_Q
                    ,coef_IPT_A, coef_IPT_K1, coef_IPT_Q
                    )

    MSEmat[S,] <- c(
        mean((    ..betaA +      ..bint*Data[,'Qvar'] +       ..bint2*Data[,'K1'] -
             (coef_DW1_A + coef_DW1_Q*Data[,'Qvar'] + coef_DW1_K1*Data[,'K1']))^2)
      ,mean((    ..betaA +      ..bint*Data[,'Qvar'] +       ..bint2*Data[,'K1'] -
             (coef_DW2_A + coef_DW1_Q*Data[,'Qvar'] + coef_DW2_K1*Data[,'K1']))^2)
      ,mean((    ..betaA +      ..bint*Data[,'Qvar'] +       ..bint2*Data[,'K1'] -
             (coef_DW3_A + coef_DW3_Q*Data[,'Qvar'] + coef_DW3_K1*Data[,'K1']))^2)
      ,mean((    ..betaA +      ..bint*Data[,'Qvar'] +       ..bint2*Data[,'K1'] -
             (coef_DW4_A + coef_DW4_Q*Data[,'Qvar'] + coef_DW4_K1*Data[,'K1']))^2)
      ,mean((    ..betaA +      ..bint*Data[,'Qvar'] +       ..bint2*Data[,'K1'] -
             (coef_OLS_A + coef_OLS_Q*Data[,'Qvar'] + coef_OLS_K1*Data[,'K1']))^2)
      ,mean((    ..betaA +      ..bint*Data[,'Qvar'] +       ..bint2*Data[,'K1'] -
             (coef_IPT_A + coef_IPT_Q*Data[,'Qvar'] + coef_IPT_K1*Data[,'K1']))^2)
      ,mean(Data[,'visit'])
      )

    visitMat[S,] <- c(mean(Data[,'visit']) ,mean(Data[,'A'])
      ,mean(Data[Data[,'visit']==1,'A']))

} ## End loop for one simulation
```

```
    catn <- function(...) cat(..., fill=T)
    catn("Mean MSE:")
    print(apply(MSEmat,2,mean))
    catn("\nMean/SD coef estimates:")
    print(rbind(apply(coefMat,2,mean),
                apply(coefMat,2,sd)))
    catn("\nMean/SD visit and exposure:")
    print(rbind(apply(visitMat,2,mean),
                apply(visitMat,2,sd)))

    invisible(list(coefMat=coefMat, MSEmat=MSEmat, visitMat=visitMat))

} ## End function



##########################################################################################

## Running times for sample sizes of 250, 500, 50000
 ## for 1 simulation

## loop is the time to create the dataset
## ps is the time to fit the weight models
## glm is the time to fit all the estimators (to obtain the blips)

>   simul.new(250,1, T, 1)
vh: 1
loop:Time difference of 0.02693295 secs
ps:  Time difference of 0.6380501 secs
glm: Time difference of 0.08373785 secs


>  simul.new(500,1, T, 1)
vh: 1
loop:Time difference of 0.06881285 secs
ps:  Time difference of 1.317435 secs
glm: Time difference of 0.2214382 secs


>  simul.new(25000,1, T, 1)
vh: 1
loop:Time difference of 2.217079 secs
ps:  Time difference of 1.69475 mins
glm: Time difference of 10.51362 secs



###########################################################################################
```

Supplementary Material F

Flow chart in the application to the CPRD, United Kingdom, 1998-2017

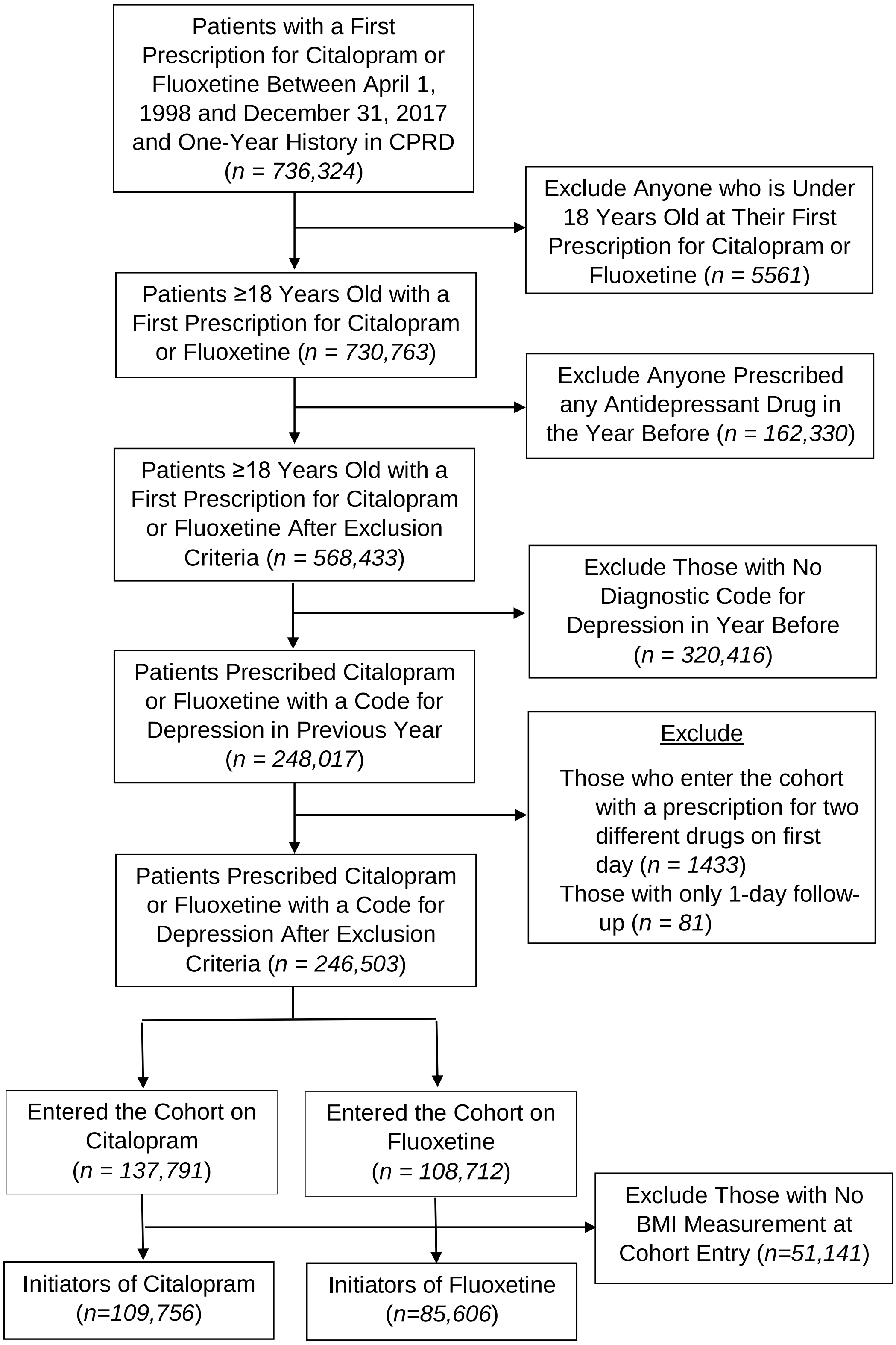

Supplementary Material G

Baseline characteristics of the study cohort, observation rate ratios for the outcome, and estimated individualized treatment rules in the application to CPRD

Supplementary Table 8: Baseline characteristics of the two study cohorts stratified by treatment at cohort entry, frequencies (%, unless otherwise noted), CPRD, UK, 1998-2017

Cohort:	BMI observed at/before cohort entry		$\geq$ 1 BMI after cohort entry	
Treatment:	Citalopram	Fluoxetine	Citalopram	Fluoxetine
Variable	(n=109,756)	(n=85,606)	(n=18,671)	(n=12,449)
Age, mean (SD)	44.2 (17.6)	41.7 (15.9)	48.5 (18.1)	45.1 (16.5)
Male sex	37,211 (34)	26,687 (31)	5965 (32)	3609 (29)
Index of Multiple Deprivation, mean (SD)	3.0 (1.4)	3.0 (1.4)	3.0 (1.4)	3.1 (1.4)
Calendar year				
1998-2005	30,341 (28)	43,553 (51)	3751 (20)	4896 (39)
2006-2011	53,470 (49)	31,481 (37)	10,279 (55)	5703 (46)
2012-2017	25,945 (24)	10,572 (12)	4641 (25)	1850 (15)
BMI at cohort entry (BMI(0)), mean (SD)	26.3 (5.7)	26.4 (5.8)	27.7 (6.6)	28.1 (6.9)
Time from measurement of BMI(0) to cohort entry in days, mean (SD)	926 (1278)	928 (1209)	627 (1060)	646 (1053)
Ever smoker	59,030 (60)	44,700 (61)	11,586 (62)	8017 (64)
Alcohol abuse	8565 (8)	5509 (6)	1478 (8)	869 (7)
Psychiatric disease†	2691 (2)	1773 (2)	521 (3)	321 (3)
Anxiety	34,216 (31)	19,437 (23)	5956 (32)	2987 (24)
Medication				
Antipsychotics	14,370 (13)	10,110 (12)	2836 (15)	1675 (13)
Other psychotropic drugs‡	23,801 (22)	16,005 (19)	4476 (24)	2546 (20)
Lipid lowering drugs	10,060 (9)	5141 (6)	3360 (18)	1614 (13)
Number of psychiatric hospitalisations in previous 6 months, mean (SD)	0.04 (0.24)	0.02 (0.30)	0.04 (0.24)	0.03 (0.34)
Characteristics measured after baseline				
First BMI after cohort entry, mean (SD)	-	-	27.9 (6.8)	28.3 (7.1)
Time in days from cohort entry to first BMI during follow-up, mean (SD)	-	-	120 (120)	110 (113)

Abbreviations: BMI, body mass index; CPRD, Clinical Practice Research Datalink; UK, United Kingdom; SD, standard deviation.

†. Indicator for diagnosis of autism spectrum disorder, obsessive compulsive disorder, bipolar disorder, or schizophrenia.

‡. Which include benzodiazepine drugs, anxiolytics, barbiturates and hypnotics.

Supplementary Table 9: Estimated rate ratios (95% bootstrap CIs) from the proportional rate model for the observation of BMI, CPRD, UK, 1998-2017, n=195,362 individuals.

Variable	Rate ratio (Bootstrap 95% CI)
Antidepressant drug = citalopram	0.94 (0.92, 0.97)
Age	1.00 (1.00, 1.00)
Male sex	0.91 (0.89, 0.93)
Index of Multiple Deprivation	1.02 (1.01, 1.03)
Calendar year (Ref.= <2006)	
2006-2011	0.93 (0.91, 0.96)
2012-2017	0.89 (0.87, 0.92)
BMI at baseline	1.02 (1.01, 1.02)
Ever smoker	1.68 (1.64, 1.72)
Alcohol abuse	1.02 (0.94, 1.11)
Psychiatric disease†	0.99 (0.86, 1.14)
Anxiety	1.01 (0.97, 1.04)
Medication	
Antipsychotics	1.10 (1.02, 1.18)
Other psychotropic drugs‡	1.23 (1.18, 1.28)
Lipid lowering drugs	1.16 (1.12, 1.19)
Number of psychiatric hospitalisations in previous 6 months	1.00 (0.97, 1.03)

Abbreviations: BMI, body mass index; CI, confidence interval; CPRD, Clinical Practice Research Datalink; UK, United Kingdom; IMD, Index of Multiple Deprivation.

†. Indicator for diagnosis of autism spectrum disorder, obsessive compulsive disorder, bipolar disorder, or schizophrenia.

‡. Which include benzodiazepine drugs, anxiolytics, barbiturates and hypnotics.

Supplementary Table 10: Coefficients of the blip function (95% bootstrap CIs) for the optimal treatment rules as estimated by four alternative models: OLS which does not adjust for confounding or observation process, IPW which accounts only for confounding, IIV which accounts only for the observation process, and the proposed doubly-weighted estimator which accounts for both processes, CPRD, UK, 1998-2017, n=31,120 individuals.

Variable	$\hat{\psi}_{OLS}$	$\hat{\psi}_{IPT}$	$\hat{\psi}_{IIV}$	$\hat{\psi}_{DW}$
Intercept	-1.46 (-2.41, -0.54)	-1.22 (-2.17, -0.31)	-1.42 (-2.33, -0.56)	-1.26 (-2.21, -0.34)
Age	0.01 (0.00, 0.03)	0.01 (0.00, 0.03)	0.01 (0.00, 0.03)	0.01 (0.00, 0.03)
Male sex	-0.08 (-0.61, 0.44)	-0.08 (-0.65, 0.46)	-0.03 (-0.53, 0.46)	-0.02 (-0.64, 0.51)
IMD	0.08 (-0.11, 0.25)	0.06 (-0.12, 0.25)	0.06 (-0.13, 0.24)	0.06 (-0.13, 0.23)
Ever smoker	0.03 (-0.46, 0.50)	-0.03 (-0.56, 0.50)	0.01 (-0.46, 0.51)	-0.04 (-0.60, 0.47)
Alcohol abuse	1.05 (0.00, 2.21)	0.75 (-0.28, 1.76)	0.84 (-0.10, 1.82)	0.46 (-0.49, 1.37)
Psychiatric disease†	0.20 (-1.65, 2.13)	0.86 (-1.11, 2.81)	0.58 (-1.13, 2.41)	1.36 (-0.46, 3.48)
Anxiety	0.49 (-0.06, 1.00)	0.47 (-0.10, 1.00)	0.50 (-0.06, 1.00)	0.46 (-0.17, 1.03)
Medication				
Antipsychotics	-0.49 (-1.21, 0.23)	-0.59 (-1.40, 0.17)	-0.52 (-1.24, 0.20)	-0.66 (-1.51, 0.15)
Other psychotropic drugs‡	-0.14 (-0.70, 0.43)	-0.04 (-0.61, 0.57)	0.11 (-0.50, 0.62)	0.24 (-0.34, 0.84)
Lipid lowering drugs	-0.02 (-0.68, 0.62)	-0.03 (-0.74, 0.61)	0.07 (-0.61, 0.75)	0.06 (-0.70, 0.81)

Abbreviations: CI, confidence interval; CPRD, Clinical Practice Research Datalink; UK, United Kingdom; IMD, Index of Multiple Deprivation.

†. Indicator for diagnosis of autism spectrum disorder, obsessive compulsive disorder, bipolar disorder, or schizophrenia.

‡. Which include benzodiazepine drugs, anxiolytics, barbiturates and hypnotics.

Supplementary Material H

Blip function evaluated under different patient profiles of characteristics (first table) and proportion of subjects who were recommended citalopram under the different estimators that were compared (second table).

Supplementary Table 11: Blip value under different patient profiles (Age set to 45 years old), CPRD, United Kingdom, 1998-2017. A dot represents a 0 (i.e., a characteristic that is not present).

Male sex (yes)	IMD (1 to 5)	Ever smoker (yes)	Alcohol abuse (yes)	Psychiatric diagnosis (yes)	Anxiety (yes)	Antipsy. drug (yes)	Psychotro. drug (yes)	Lipid lowering drug (yes)	Value blip function
1	1	·	·	·	·	1	·	·	-1.43
·	1	·	·	·	·	1	·	·	-1.41
1	3	·	·	·	·	1	1	1	-1.02
·	3	·	·	·	·	1	1	1	-1.00
1	1	·	·	·	·	·	·	·	-0.78
·	1	·	·	·	·	·	·	·	-0.76
1	3	1	·	·	·	·	·	·	-0.71
·	3	1	·	·	·	·	·	·	-0.69
1	5	·	·	·	·	·	·	·	-0.55
·	5	·	·	·	·	·	·	·	-0.53
1	3	1	1	·	·	·	·	·	-0.24
·	3	1	1	·	·	·	·	·	-0.22
1	3	·	·	·	1	·	·	·	-0.21
·	3	·	·	·	1	·	·	·	-0.19
1	5	1	1	·	·	·	·	·	-0.13
·	5	1	1	·	·	·	·	·	-0.11
1	3	1	1	1	·	·	·	·	1.12
·	3	1	1	1	·	·	·	·	1.13
1	3	·	1	1	·	·	·	·	1.16
·	3	·	1	1	·	·	·	·	1.18
1	3	1	·	1	1	·	·	1	1.18
·	3	1	·	1	1	·	·	1	1.20
1	5	1	1	1	·	·	·	·	1.23
·	5	1	1	1	·	·	·	·	1.25
1	3	·	1	1	1	·	·	·	1.62
·	3	·	1	1	1	·	·	·	1.64
1	5	1	1	1	1	·	1	1	1.99

Supplementary Table 11 shows several profiles of individuals and the corresponding estimates

of the blip function found using $\hat{\psi}_{DW}$ from our proposed approach. The sign of the estimated blip function indicates which treatment is to be recommended. For instance, a male with an Index of Multiple Deprivation of 1 who never smoked, had no alcohol abuse, no diagnosis for psychiatric diseases, no anxiety diagnosis, and who used antipsychotic drugs but did not use other psychotropic drugs or lipid-lowering drugs obtains the lowest blip value of -1.43 and, therefore, his recommended treatment is fluoxetine. A male with an Index of Multiple Deprivation of 5 who is an ever smoker, who had alcohol abuse, received a diagnosis for psychiatric disease, received a diagnosis for anxiety, did not use antipsychotic drugs but used other psychotropic drugs and lipid-lowering drugs obtains a blip value of 1.99 and, therefore, his recommended treatment is citalopram.

Supplementary Table 12: Proportion of patients' records corresponding to a recommendation for citalopram under different estimation strategies, CPRD, United Kingdom, 1998-2017, $n = 47,938$ records

Estimator	Proportion (%)
$\hat{\psi}_{OLS}$	8.3
$\hat{\psi}_{IPT}$	10.9
$\hat{\psi}_{IIV}$	7.7
$\hat{\psi}_{DW}$	8.7

Supplementary Table 13: Comparison of the recommendations across the different estimation strategies (C, citalopram, F, fluoxetine), CPRD, United Kingdom, 1998-2017, $n = 47,938$ records

		$\hat{\psi}_{IPT}$		$\hat{\psi}_{IIV}$		$\hat{\psi}_{DW}$	
		F	C	F	C	F	C
$\hat{\psi}_{OLS}$	F	42,156	1780	43,434	502	41,975	1961
$\hat{\psi}_{OLS}$	C	563	3439	815	3187	1789	2213
$\hat{\psi}_{IPT}$	F			42630	89	42203	516
$\hat{\psi}_{IPT}$	C			1619	3600	1561	3658
$\hat{\psi}_{IIV}$	F					42680	1569
$\hat{\psi}_{IIV}$	C					1084	2605
